# Proangiogenic Effect of Affinin and an Ethanolic Extract from *Heliopsis longipes* Roots: Ex Vivo and In Vivo Evidence

**DOI:** 10.3390/molecules26247670

**Published:** 2021-12-18

**Authors:** Paola Estefanía García-Badillo, Anaguiven Avalos-Soriano, Josué López-Martínez, Teresa García-Gasca, Jesús Eduardo Castro-Ruiz

**Affiliations:** 1 Laboratorio de Biología Celular y Molecular, Facultad de Ciencias Naturales, Universidad Autónoma de Querétaro, Querétaro 76230, Querétaro, Mexico; paoegb@hotmail.com (P.E.G.-B.); francisco.josue.lopez@uaq.mx (J.L.-M.); 2 Cátedras CONACYT-Centro de Investigación en Alimentación y Desarrollo, A.C., Av. Sábalo Cerritos S/N, S/C, Mazatlán 82112, Sinaloa, Mexico; anaguiven.avalos@ciad.mx; 3 Escuela de Odontología, Facultad de Medicina, Universidad Autónoma de Querétaro, Querétaro 76176, Querétaro, Mexico

**Keywords:** affinin, chilcuague, *Heliopsis longipes*, angiogenesis, aortic rings, angioreactor

## Abstract

Angiogenesis, the formation of new blood vessels, underlies tissue development and repair. Some medicinal plant-derived compounds can modulate the angiogenic response. *Heliopsis longipes*, a Mexican medicinal plant, is widely used because of its effects on pain and inflammation. The main bioactive phytochemicals from *H. longipes* roots are alkamides, where affinin is the most abundant. Scientific studies show various medical effects of organic extracts of *H. longipes* roots and affinin that share some molecular pathways with the angiogenesis process, with the vasodilation mechanism of action being the most recent. This study investigates whether pure affinin and the ethanolic extract from *Heliopsis longipes* roots (HLEE) promote angiogenesis. Using the aortic ring rat assay (ex vivo method) and the direct in vivo angiogenesis assay, where angioreactors were implanted in CD1 female mice, showed that affinin and the HLEE increased vascular growth in a dose-dependent manner in both bioassays. This is the first study showing the proangiogenic effect of *H. longipes*. Further studies should focus on the mechanism of action and its possible therapeutic use in diseases characterized by insufficient angiogenesis.

## 1. Introduction

Alkamides are bioactive compounds contained in at least 33 plant families, composed by the union of an amine and a medium-to-long-chain fatty acid, generally aliphatic, through an amide linkage. Depending on the type of unsaturated bonds, they are classified into olefinic alkamides with at least one double bond and acetylenic alkamides with at least one triple bond. The importance of alkamides lies in the fact that they are bioactive compounds that evoke a remarkable response in receptor cells [1,2,3]. Another set of alkamides have homo- or heterocyclic rings at the chain terminal extreme, structures observed in the capsaicin from some chili peppers (*Capsicum* spp.) and the piperine in peppers (*Piper* spp.) [2,4,5]. Alkamides are structurally related to endogenous compounds in mammals, the endocannabinoids. N-arachidonoylethanolamine (anandamide), an endogenous cannabinoid brain neurotransmitter with remarkable structural similarity to plant alkamides, has its greatest activity in the central nervous system, where it exhibits its biological effects [3].

**Figure 1:**
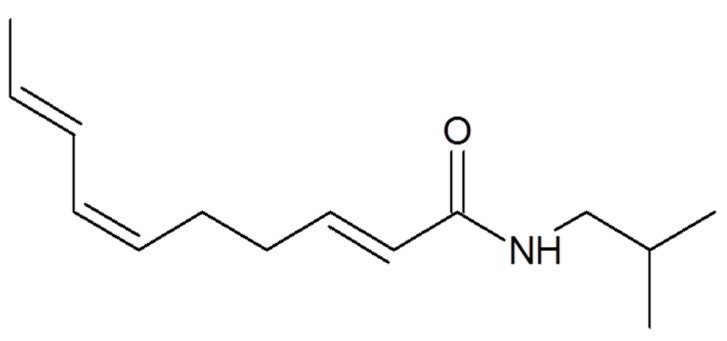
Chemical structure of affinin.

Mexico has around 4000 species of plants with medicinal attributes, of which only 5% have been scientifically evaluated [6]. *Heliopsis longipes* S.F. Blake is a native plant from the central region of Mexico, with roots colloquially known as “chilcuague” [7] that are commonly used as condiments or an analgesic [8]. The main phytochemical in *H. longipes* roots is a bioactive alkamide denominated affinin [9,10], an unsaturated aliphatic isobutylamide (N-isobutyl-2E, 6Z, 8E-decatrienamide) (Figure 1), also known as spilanthol, which was isolated in 1945 [11,12] and is present in species belonging to the *Heliopsis*, *Wedelia*, and *Spilanthes* genera in the Asteraceae family [11,13,14,15].

Affinin and different organic extracts of *H. longipes* roots are responsible for observed biological effects such as local anesthetic, flavoring, insecticidal and larvicidal [16], antimicrobial, bactericidal, and fungistatic effects [17,18]. The pharmacological effects of affinin were evidenced as an anti-inflammatory in a macrophage cell line [19] and in rodents [20], antinociceptive in mice [21,22], anxiolytic and diuretic in mice [23], and antihyperalgesic [24]. Safety tests evaluated the acute toxicity in mice, and the mean lethal dose (LD50 = 113 mg/kg) was significantly higher compared to the dose required to obtain antinociception. Furthermore, no mutagenic effects were found by the Ames test [25], and the antimutagenic effects of affinin were observed [8]; cytotoxic effects were not reported [19].

The most studied pharmacological effect of affinin and *H. longipes* extracts is antinociception [21,22,24,25]. Animal studies showed a concentration-dependent analgesic effect with comparable potency to widely used drugs such as morphine and nonsteroidal anti-inflammatory drugs (NSAIDs) [22]. Studies focused on elucidating the possible mechanism of action of affinin, suggesting its interaction with several signaling pathways such as the gamma-aminobutyric acid (GABA) pathway, serotonin, opioids, the nitric oxide (NO) pathway, and the activation of potential transient receptor (TRP) channels, specifically TRPV1 as a potential analgesic treatment for orofacial pain [22,26]. Animal nociception tests, in the presence of pharmacological inhibitors glibenclamide and 1H-[1,2,4]oxadiazolo [4,3-a]quinoxalin-1-one (ODQ), demonstrated that affinin activates the NO/PKG/KATP signaling pathways to elicit an antinociceptive effect [22].

Affinin is able to permeate the oral mucosa and skin [11,27] and the blood-brain barrier [28]. Nevertheless, information about the interaction between alkamides and vasculature is scarce. Recently, we reported that affinin and *H. longipes* dichloromethane and ethanolic extracts promoted vasodilation in rat aortic rings, where concentration-dependent vasodilation in isolated rat aorta segments was observed [29]. The affinin vasodilator effect on aorta rings was inhibited in the presence of NG-nitro L-arginine methyl ester (L-NAME), cystathionine-γ-lyase (CSE), tetraethylammonium (TEA), and ODQ, indicating that in the vasculature, affinin induces the NO/cGMP and H_2_S/KATP signaling pathways. These results confirmed the participation of gasotransmitters (GTs) (nitric oxide, NO; hydrogen sulfur, H_2_S; and carbon monoxide, CO) and prostacyclin (PGI2) pathways. The signaling pathways involved in NO-mediated vasodilation also parallelly participate in the angiogenesis process [30,31,32,33]. Moreover, affinin activates TRPV1 to induce antinociception [26], and it produces vasodilation, interacting with endothelial TRV1 and TRPA1 channels with the subsequent activation of the NO/cGMP, H_2_S/KATP, and HNO/TRPA1/CGRP signaling pathways [34]. Nitroxyl (HNO) is a gasotransmitter that activates TRPA1 channels with the subsequent release of CGRP, a neuropeptide mainly present in nervous tissue, endothelial, and bone cells, which plays an important role in bone remodeling and angiogenesis [35,36].

The interaction between affinin and TRP channels is an interesting field for pharmacological research, as the same molecular pathways are implied in angiogenic activity, the formation of new blood vessels from existing ones. This process is also the result of the net balance between positive and negative regulators affected under multiple diseases, including cancer, vasoproliferative retinopathies, rheumatoid arthritis, diabetic ulcers, and cardiovascular diseases [37]. The angiogenesis signaling cascade provokes an increase in calcium within endothelial cells, which increases NO via the endothelial NO synthase isoform (eNOS), resulting in augmented vascular permeability. The major signaling pathway for NO includes binding to soluble guanylyl/guanylate cyclase (sGC) and the production of the second messenger, cyclic guanosine monophosphate (cGMP), followed by the activation of protein kinase G (PKG). In addition, the vascular hydrogen sulfide (H_2_S) pathway, generated from L-cysteine, activates phosphatidylinositol-3-OH-kinase–protein kinase B (PI3K–Akt), increasing eNOS phosphorylation and vascular permeability. Once the signaling cascade activates PKG, extracellular signal-regulated kinases (Erk 1 and 2) are phosphorylated, enhancing proliferation and migration to new blood vessels. When the mitogen-activated protein kinase (p38 MAP) is phosphorylated, it increases vascular permeability and acts as a regulator of inflammation [30,33,38,39,40,41].

This evidence suggests that affinin is a promising natural product that can treat diseases, including those with an impaired wound-healing process. Here, for the first time, we report the proangiogenic effects of *H. longipes* affinin and ethanolic alkamide extract by in vivo and ex vivo assays. This investigation represents the first screening of the novel biological activity of affinin as a proangiogenic molecule.

## 2. Results

**Figure 2:**
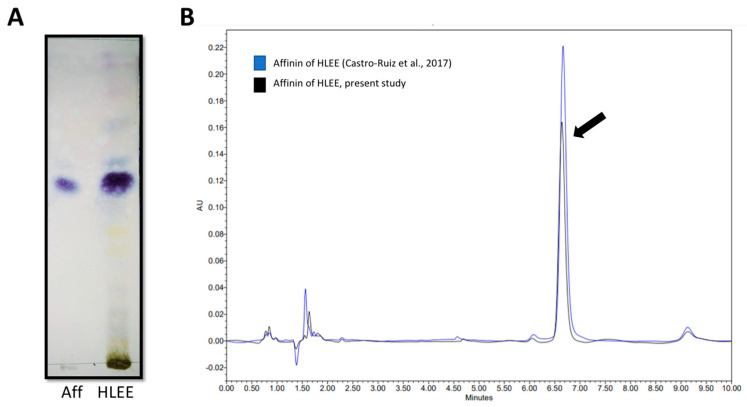
Chromatographic analysis of an ethanolic extract of *H. longipes* (HLEE) and affinin. (**A**) TLC showing affinin (Aff) compared to ethanolic extract (HLEE). (**B**) HPLC shows the presence of affinin (arrow) in HLEE.

The extraction process for the *H. longipes* ethanolic extract (HLEE) resulted in a yield of 54.7 g/kg roots dry weight. Chromatographic analysis, thin-layer chromatography (TLC), and high-performance liquid chromatography (HPLC) confirmed that affinin was the most abundant phytochemical in the extract (Figure 2). Under our experimental conditions, the addition of UAE only increased the whole extract yield without improving the affinin amount when compared with our previous study [29].

**Figure 3:**
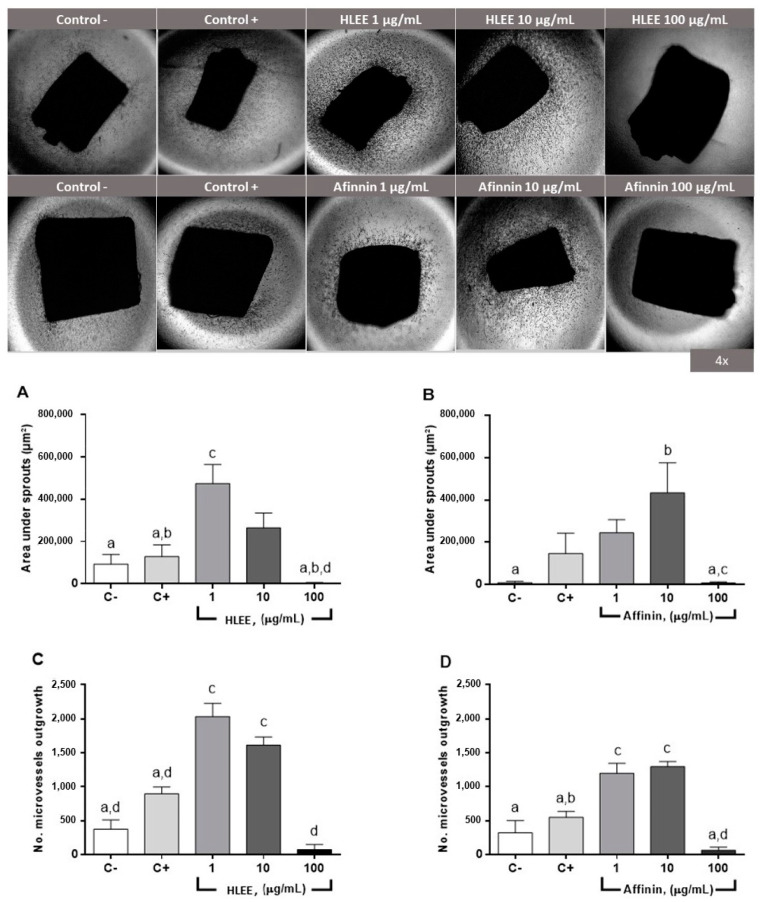
Proangiogenic effect of HLEE and affinin on isolated rat aorta rings. (**top**) Representative micrographs of aortic rings that were untreated (Control−, C−), VEGF-treated (Control+, C+), and treated with different concentrations of HLEE or affinin. (**bottom**) (**A**,**B**) Evaluation of migration area for HLEE and affinin, respectively, and (**C**,**D**) number of new blood vessel cellular outbreaks for HLEE and affinin, respectively. Values are expressed as mean ± standard error of the mean (SEM) (*n* = 9). Small letters represent statistically significant difference (Tukey, *p* < 0.05).

**Figure 4:**
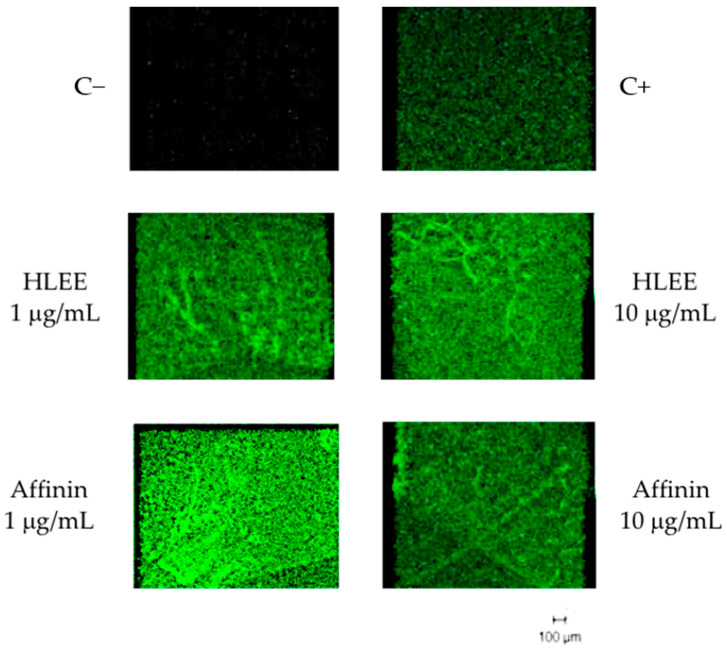
Immunofluorescence staining of rat aortic rings. Confocal images of aortic rings embedded in collagen and stained with BS1 lectin-FITC (green) 10 days after embedding. Positive control was treated with 30 ng/mL FGFb. Scale bar, 100 µm.

Angiogenesis was determined using rat aortic ring assay, where HLEE and purified affinin stimulated concentration-dependent angiogenesis evaluated as the migration area (µm^2^) and number of new outbreaks (Figure 3). The maximal effect on the migration area and the number of sprouts occurred at 1 and 10 µg/mL for HLEE and affinin, respectively. No effect was observed at the highest tested concentration (100 µg/mL). Fluorescence staining with FICT-lectin identified the presence of endothelial cells only after treatment with HLEE and affinin (Figure 4).

**Figure 5:**
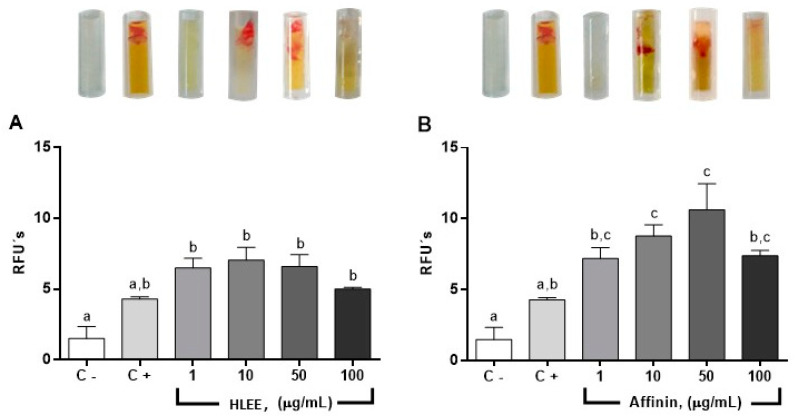
Evaluation of the angiogenic effect of (**A**) HLEE and (**B**) affinin using DIVAA experimental protocol. Blood vessels are measured into angioreactors by relative fluorescent units (RFUs) of vascular cells stained with fluorescent isolectin-B4. Values compared with a negative control without VEGF + bFGF (C−) and angioreactors containing only VEGF + bFGF (positive control, C+). Values are mean ± SEM, *n* = 4. Letters on bars (a, b, c, d) represent a statistically significant difference, *p* < 0.05.

A direct in vivo angiogenesis assay (DIVAA) showed no signs of toxicity, suggesting the safety of both treatments, HLEE and affinin. This assay consisted of measuring the invasion of blood vessels into subcutaneously implanted cylinders containing a basement membrane mixed or not with VEGF (angioreactors). The presence of vascular tissue measured by the relative fluorescent units (RFUs) of vascular cells stained by fluorescent isolectin-B4, a marker of endothelial cells, showed that affinin and HLEE induced a significant angiogenic effect at 10 and 50 µg/mL, respectively (Figure 5) compared to the negative control (C−). However, the effect of both compounds decreased at the highest tested concentration (100 µg/mL). These results are consistent with those of the ex vivo assay.

## 3. Discussion

For the first time, the present study shows that affinin and HLEE exhibited proangiogenic effects. *Heliopsis longipes* roots were widely studied for medicinal purposes, where a variety of extraction processes and analytical methods were used to study the principal phytochemicals, alkamides [20,21,22,24,25,42,43]. Regarding the extraction process, maceration in combination with UAE enhanced the HLEE yield threefold (54.7 g/kg roots dry weight), with respect to previous reports that used maceration extraction alone (17 g/kg roots dry weight) [29]. TLC and HPLC chromatography showed that the main phytochemical in HLEE was affinin. However, despite the enhanced amount of HLEE being obtained, no increase in affinin extraction was found, suggesting that UAE favors the extraction of phytochemicals other than affinin.

Numerous assays were developed to evaluate the angiogenic process in animal models [44,45]. Rat aortic ring assay has many advantages: it is inexpensive, few animals are required to obtain a significant sample, and the development of new vascular sprouts can be followed by representative images of the angiogenesis steps, making it possible to successfully evaluate angiogenesis inhibitors and analyze possible molecular factors involved [44,45,46]. The main limitation of this assay is that it is not possible to evaluate the interaction of angiogenesis and other processes as in an in vivo model [47]. This method made it possible to determine that affinin and HLEE induced new blood vessel sprouts at 1 and 10 µg/mL; however, when using 100 µg/mL, an inhibitory effect was observed.

In order to observe the in vivo response, DIVAA was used. The use of angioreactors offers a practical and sensitive assay of angiogenesis. It is highly suited to identify pro-and antiangiogenic agents by delimiting a stable volume of the sample, maintaining its structure (directing the growth of the blood vessels), using a smaller number of reagents than that in other in vivo assays [48,49,50,51]. It is a reproducible and quantitative model and allows for the retrieval of the material contained in the assay for more biochemical, cellular, or genetic tests. However, inadequate handling of the material may provoke the premature gelling of the BME, and the devices must be appropriately implanted to decrease result variability [31,48]; therefore, special training is needed to use this method. This model is thus extensively used to evaluate the pro-and antiangiogenic potential of plant extracts and purified compounds [52,53,54]. We used this model instead of an in vitro model because it offers more information in more complex conditions, such as a living organism, where the interaction between biotic and abiotic factors can affect the angiogenic effect of affinin or HLEE. These tests also allowed for observing the cellular environment within a matrigel matrix through an inverted microscope, distinguishing the new blood vessels’ morphology and following its evolution [49,55,56]. Isolectin-B4 conjugated with fluorescein isothiocyanate (FITC), lectin I from *Griffonia Simplicifolia* labeled with fluorescence that binds to alpha-D-galactosyl and N-acetyl galactosaminyl groups on the surface of endothelial cells, allowing for the detection of endothelial cells in both ex vivo and in vivo tests [48,57,58].

Our results showed a proangiogenic effect of affinin and HLEE from 1 to 50 µg/mL, but 100 µg/mL exhibited an inhibitory effect in the rat aorta ring assay. VEGF and bFGF were used as positive controls because they exhibited a proangiogenic effect in different studies [38,52,59,60,61,62]. The proangiogenic effect of HLEE and affinin was higher than that of the positive control in both ex vivo and the in vivo experiments, even in the fluorescence experiment. The ex vivo assay using rat aorta rings showed that HLEE exhibited the highest effect at a concentration of 1 µg/mL; for affinin, this was at 10 µg/mL. However, the in vivo assay showed that the highest observed effect was for affinin at 50 µ/mL, suggesting that the observed effect mainly depends on this alkamide, even though alkamide-based extracts enhanced the pharmacological response to the pure compound, strongly suggesting that alkamides could have a synergic biological response [16,21,22,28,42,43,63].

The absence of toxic effects in HLEE and affinin in the in vivo study is consistent with doses used in previous studies [22,25,29]. The effects on angiogenesis were similar to those found for *N*-arachidyl ethanolamine (anandamide), an endogenous compound of the same class of endocannabinoids, structurally related to alkamides [64,65,66,67]. Other investigations with alkamides, such as on *Acmella oleracea* crude extract (15%) and *Achyrocline satureioides* essential oil (1.5%), showed a beneficial effect on wound healing that may involve increased angiogenesis and its potential therapeutics in tissue repair [68]. Dermane and Passaro [69] also showed the effect of subcutaneous muscle contraction produced by *Acmella oleracea* extract. In addition, a positive effect of dry hydroalcoholic extract of *Echinacea purpurea* on the angiogenic activity of human blood mononuclear cells was shown, meaning prospective use in patients with systemic scleroderma, coronary heart disease, and some patients with oral candidiasis.

Scientific information about the interaction between alkamides and vasculature is scarce. In vivo and in vitro experiments have supported that pellitorine and affinin can permeate oral mucosa, a well-known vascularized tissue, and thus be absorbed into the bloodstream [28,70]. The transmucosal pathway is a versatile pharmacological route because the drugs are not subjected to first-step metabolism. Therefore, through the oral mucosa, a large amount of the absorbed drug will be directly conducted to the intern jugular and peripheral vessels [11]. Moreover, affinin and pellitorine administrated *per orem* could be absorbed by the intestinal mucosa, and thus, be present in systemic blood circulation [28,70]. Moreover, a previous study reported that affinin and pellitorine could cross the blood-brain barrier (BBB), proving that those alkamides interact with BBB endothelial cells and permeate the cerebral cortex parenchyma to produce pharmacological effects at the central nervous system level [21,25,28,43,70,71]. Accordingly, it is likely to presume that affinin could produce some effect on blood vessels.

Recently, we reported that affinin could induce vasodilation in rat aorta with a mechanism that includes the activation of the main GTs (NO, H_2_S, and CO), potassium channels in an endothelium partially-dependent effect [29]. The activation of eNOS, cystathionine gamma-lyase (CSE), and heme oxygenase 1 (HO-1) proteins result in the synthesis of NO, carbon monoxide (CO), and hydrogen sulfide (H_2_S), respectively, widely known members of the GTs family. These compounds play a pivotal role in vasodilation and angiogenesis [30]. Within the molecular mechanism of the angiogenic effect, the activation of the NO signaling pathway is suggested, and other GTs in the vasodilator effect of affinin [29]. Supporting this hypothesis, there is evidence of the central participation of GTs in angiogenesis [30,72]. Another work revealed that the molecular targets of affinin in rat aorta endothelium are TRPV1 and TRPA1 channels, as well as cannabinoids receptors [34]. Affinin and other isobutylamides target TRPV1 and TRPA1 channels to produce pharmacological effects [26,73,74,75]. There is scientific evidence that TRPV1 and TRPA1 are highly expressed in the vasculature, including in the endothelium [76,77,78,79]. The evidence of the vasodilator effect induced by affinin involving the activation of TRPA1 and TRPV1 channels and CB1 and eCB receptors was complemented by in silico analyses that showed high-affinity binding between affinin and the receptors. Moreover, anandamide produces a potent vasodilator effect through mechanisms that include the activation of transient potential vanilloid 1 (TRPV1) channels and cannabinoid receptors that activate the NO/cGMP pathway [66].

The vasodilatory effect of affinin is also involved in the HNO-TRPA1-CGRP pathway [34]. It has been stated that, in the endothelium, NO and H_2_S react to form a sibling gaseous compound denominated nitroxyl (HNO), which in turn, can activate TRPA1 channels in the perivascular nerve endings, generating the release of Calcitonin Gene-Related Peptide (CGRP), a potent vasodilator and angiogenic neuropeptide [35,36,72,80,81]. Therefore, affinin-induced vasodilation is partly mediated via activation of the HNO–TRPA1–CGRP pathway [34].

Here, we report the proangiogenic effect of *H. longipes* roots alkamides. We contributed scientific evidence that helps to support our hypothesis that such an effect may be closely related to the previously reported vasodilator molecular pathways. It is important to take into account that affinin can cross epithelial barriers like skin and mucosa, to reach the vasculature and that it can activate endothelial molecular targets like TRPV1 and TRPA1 channels, that produce a rise in intracellular Ca^2+^ and the activation of the GTs signaling pathway, mechanisms involved in the stimulation of angiogenesis. Further studies are needed to confirm the participation of such mechanisms in the angiogenic effect.

## 4. Materials and Methods

### 4.1. Reagents

The reagents and solvents used in the phytochemical study of the *H. longipes* roots were purchased from JT Baker (Phillisburg, NJ, USA). For pharmacological assays, reagents were obtained from Sigma-Aldrich (St. Louis, MO, USA), and a previously purified affinin aliquot was provided by the research group. The directed in vivo Angiogenesis Assay (DIVAA^®^) Starter Kit was purchased from Trevigen (Gaithersburg, MD, USA).

### 4.2. Animals

All the experiments were performed under the guidelines of the Official Mexican Standard NOM-062-ZOO-1999 for the reproduction, care, and use of laboratory animals [82], and the protocol was approved by the Committee of Bioethics of the Faculty of Natural Sciences, Autonomous University of Queretaro (registration number 10001). We used 28 Wistar male rats (250–300 g) and 24 CD-1 female mice (25–30 g) for the ex vivo and in vivo pharmacological studies, respectively. Animals were acquired at the Institute of Neurobiology of the National Autonomous University of Mexico, Campus Juriquilla, Queretaro, Qro., and housed under controlled temperature conditions, 12:12 h light-dark cycle, and providing of water and food ad libitum.

### 4.3. Plant Material

Fresh *H. longipes* roots were obtained from a local supplier and identified by comparison with two complete specimens from the ethnobotanical collection (*H. longipes* vouchers J.E. Castro R.1. and R.2.) of the Jerzy Rzedowski Herbarium of the Faculty of Natural Sciences, Autonomous University of Queretaro, Qro.

### 4.4. Extraction

The dried and ground roots of *H. longipes* were macerated with absolute ethanol (JT Baker, Phillisburg, NJ, USA) for a week at a 1:10 w/v ratio and subjected to ultrasound-assisted extraction (UAE) using a sonicator (Branson 5510, Danbury, CT, USA) for 15 min at the 1st and 3rd day of maceration. The extract was filtered and then concentrated with a rotary evaporator (BÜCHI R-200, Flawil, Switzerland).

### 4.5. Pharmacological Assays

#### 4.5.1. Rat Aortic Ring Assay

The reported method by Baker et al. was slightly modified [58]. Briefly, rats were sacrificed by decapitation, and the thoracic aorta was then surgically removed. The aorta was placed into a plate containing ice-cold Krebs–Henseleit solution (126.8 nM NaCl; 5.9 nM KCl; 1.2 nM KH_2_PO_4_; 1.2 nM MgSO_4_; 5.0 nM D-glucose; 30 nM NaHCO_3_; 2.5 nM CaCl_2_), pH 7.4, 4 °C and oxygenated with carbogen (95% O_2_ and 5% CO_2_); the intraluminal space was rinsed with fresh solution to avoid clot formation. The vessel was then cleaned by removing the surrounding connective and adipose tissue, sectioning into rings from 1 to 2 mm in length, and washed with fresh Krebs–Henseleit solution. Aortic rings were transferred to a plate containing chilled Hank’s balanced salt solution (HBSS) to be washed and then cultured under a laminar flow hood, as follows: a three-dimensional matrix of type I collagen gel (Advanced BioMatrix) was prepared at a concentration of 2 mg/mL, pH 7.2 and 4 °C to avoid premature collagen polymerization [44,45,59]; then, aorta rings were each seeded using 100 µL of collagen on sterile 96-well microplates and incubated at 37 °C in 5% CO_2_ for 60 min to complete the polymerization of the media. After that, aortic rings were divided into eight treatment groups (*n* = 6 per group). Individually, aortic rings received 150 µL of one of the following treatments, previously dissolved in the basal supplemental culture medium (BSCM), prepared with Opti-MEM (Gibco, cat. 11058021, Grand Island, NY, USA) culture medium, 2.5% of fetal bovine serum (FBS) (PAN Biotech, Cat. P30-3306, Aidenbach, Germany) and 1% antibiotic–antimycotic (Gibco, cat. 15240-062, NY, USA). We used 1 µL of dimethylsulfoxide (DMSO) for each 2 µg of sample for the dilution and treatment preparations for HLEE and affinin [8,83,84]. Experiments were performed using BSCM + DMSO for the negative control; BSCM + 30 ng/mL basic fibroblast growth factor (FGF-b) (Peprotech, Cat. #450-33, East Windsor, NJ, USA) as positive control; BSCM + FGF + HLEE to study 3 concentration groups of 1, 10 and 100 µg/mL; and BSCM + FGF-b + affinin at 1, 10 and 100 µg/mL. Aortic rings were incubated at 37 °C (90% O_2_ and 5% CO_2_), and the media of the different treatments were changed (130 µL) every second day for 10 days. In that period, digital images using a 4× objective were also obtained daily by a digital camera coupled to a VE-BC3 PLUS digital microscope (Velab, CO, USA) using IS Capture 3.6.8 software (ISCapture.ink). Lastly, the number of new blood vessels was quantified at day 10 (number of new vessels/objects on the periphery of the aortic ring), and the migration area of new blood flares (µm^2^) per sample, with the Image-Pro Plus program version 5.1.2.59 for Windows XP (Media Cybernetics Inc., Silver Spring, MD, USA).

#### 4.5.2. Fluorescence Staining

On the basis of the literature [58], after 10 days of incubation, the aortic rings were washed (PBS 1X) and fixed with formalin 4% for 30 min at room temperature. After another wash with PBS 1X, permeabilization was conducted with 0.5% Triton X-100 for 30 min (37 °C) under mechanical stirring using a hybridization stirrer (Amersham Pharmacia Biotech, Amersham, Buckinghamshire, UK). After that, aortic preparations were washed twice and incubated with 200× FITC-Lectin fluorophore (50 µg/mL; cat. 3450-048-06, Trevigen, Gaithersburg, MD, USA) overnight at 4 °C. The next day, two washes were performed (PBS 1X), and aortic rings of different treatment groups were mounted onto slides with coverslips. Confocal laser scan images were captured using an inverted microscope with a Zeiss LSM 510 laser scan confocal system (Carl Zeiss, Thornwood, NY, USA).

#### 4.5.3. Direct In Vivo Angiogenesis Assay (DIVAA)

The DIVAA^®^ Starter Kit (Trevigen, Gaithersburg, MD, USA) was used to evaluate the in vivo angiogenic effect of HLEE and affinin, following the methodology described by the fabricant and by Guedez et al. [48]. The kit included angioreactors (AR) (Cat. 3450-048-01, Trevigen, Gaithersburg, MD, USA), sterile cylindrical devices (measuring 1 cm in length, 3 mm in width, and 0.15 cm in internal diameter) that, under sterile conditions working with the reagents and material at 4 °C under laminar flow hood, were filled with 25 µL of the Basement Membrane Extract (BME), growth factor reduced PathClear^®^ (Trevigen, cat. 3450-048-02, Gaithersburg, MD, USA) mixed with PBS 1X for the negative control, or with FGF-2 (1.8 µg)/VEGF (600 ng; cat. 3450-048-B10, Trevigen, Gaithersburg, MD, USA) and heparin solution (cat. 3450-048-08, Trevigen, Gaithersburg, MD, USA) for the positive control. Treatments were prepared using HLEE or affinin at different concentrations (1, 10, 50, or 100 µg/mL), resulting in 8 treatment groups. After that, ARs were incubated (37 °C for 1 h) and subcutaneously implanted in the cervical area of CD-1 mice previously anesthetized using xylazine (2%)/zoletil 50 (tiletamine 2.5% and zlazepam 2.5%), one AR per mouse. The animals were kept under observation and monitored for wound healing for 12 days. Then, the animals were sacrificed, and ARs were removed with new vessels inside them or not, depending on the different groups. The ARs were observed and photographed with 4× inverted microscopy (Zeiss, Axio Vert. A1, Jena, Germany). After that, the BME was collected and digested using 300 µL of dispase solution (CellSperse^TM^; cat. 3450-048-05, Trevigen, Gaithersburg, MD, USA) for 3 h at 37 °C. The digestion product was processed according to the manufacturer’s instructions and centrifugated (5 min at 1700 rpm) (Allegra 21R, Beckman Coulter, Fullerton, CA, USA). Pellets were suspended in new DMEM with FBS 10% for 1 h, centrifugated again, washed three times (DIVAA Wash Buffer, cat. 3450-048-07, Trevigen, Gaithersburg, MD, USA), and labeled with isolectin-B4 fluorophore conjugated to fluorescein isothiocyanate (FITC; cat. 3450-048-06, Trevigen, Gaithersburg, MD, USA) in a 96-well plate. On the next day, fluorescence was determined by spectrofluorimetry Varioskan™ Flash ™ (Thermo Fisher Scientific™, Waltham, MA, USA), in Microplate Reader software SkanIt™ (Thermo Fisher Scientific™, Waltham, MA, USA) at 485 nm of excitation and 510 nm of emission. Endothelial cells were determined as relative fluorescence units (URFs).

### 4.6. Statistical Analysis

For the isolated rat aorta test, three different rats were used for each experiment in triplicate (*n* = 9). For the DIVAA, control groups and different concentrations of the tested substances were carried out using 4 different mice (*n* = 4). All values were expressed as the mean ± the standard error of the mean (SEM) and were analyzed using statistical software GraphPad Prism version 6.01 (San Diego, CA, USA) using one-way analysis of variance (ANOVA) and Tukey’s post hoc test. A 95% confidence interval was used, and values of *p* < 0.05 were considered to be significant.

## 5. Conclusions

This is the first report about the angiogenic effect of *H. longipes* ethanolic extracts and affinin, contributing to understanding its effects in the vascular system. Experimental evidence suggests that the effect is mainly produced by affinin, which represents a promising prototype for synthesizing molecules with angiogenic activity for wound healing or the treatment of cardiovascular diseases. However, more studies are needed to further understand the molecular pathways and mechanism of action.

## Data Availability

The manuscript has been read and approved by all the named authors, and there are no other persons who satisfied the criteria for authorship but are not listed. The order of authors listed in the manuscript has been approved by all authors. We have given due consideration to the protection of intellectual property associated with this work, and there are no impediments to publication, including the timing of publication, with respect to intellectual property. We have followed the regulations of our institutions concerning intellectual property. Any aspect of the work covered in this manuscript that has involved experimental animals has been conducted with the ethical approval of all relevant bodies, and such approvals are acknowledged within the manuscript. The corresponding author is the sole contact for the editorial process (including the editorial manager and direct communications with the office). They are responsible for communicating with the other authors about progress, submissions of revisions, and final approval of proofs. Current, correct email addresses from all authors have been provided, accessible by the corresponding author, and configured to accept emails.

## Sample Availability

Ethanolic extract of *Heliopsis longipes* sample is available from the authors.
